# Horizon 2020 Priorities in Clinical Mental Health Research: Results of a Consensus-Based ROAMER Expert Survey

**DOI:** 10.3390/ijerph111010915

**Published:** 2014-10-21

**Authors:** Iman Elfeddali, Christina M. van der Feltz-Cornelis, Jim van Os, Susanne Knappe, Eduard Vieta, Hans-Ulrich Wittchen, Carla Obradors-Tarragó, Josep Maria Haro

**Affiliations:** 1 Department of Health Promotion, School for Public Health and Primary Care (CAPHRI), Maastricht University, MD 6200, Maastricht, the Netherlands; 2 Tranzo Department, Tilburg University, LE 5000, Tilburg, the Netherlands; E-Mail: C.M.vdrFeltz@uvt.nl; 3 Clinical Centre for Body, Mind and Health, GGz Breburg, Tilburg, 5042 DA, the Netherlands; 4 Trimbos Institute, AS 3500, Utrecht, the Netherlands; 5 Department of Psychiatry and Psychology, South Limburg Mental Health Research and Teaching Network, Euron, Maastricht University Medical Center, MD 6200, Maastricht, The Netherlands; E-Mail: j.vanos@maastrichtuniversity.nl; 6 Institute of Clinical Psychology and Psychotherapy, Technische Universität Dresden, Dresden 01062, Germany; E-Mails: knappe@psychologie.tu-dresden.de (S.K.); wittchen@psychologie.tu-dresden.de (H.-U.W.); 7 Institute of Neuroscience, Hospital Clinic, University of Barcelona, IDIBAPS, CIBERSAM, Barcelona 08036, Spain; E-Mail: EVIETA@clinic.ub.es; 8 Centro de Investigación Biomédica en Red de Salud Mental, CIBERSAM, Madrid 28029, Spain; E-Mails: c.obradors@pssjd.org (C.O.-T.); jmharo@pssjd.org (J.M.H.); 9 Research and Development Unit, Parc Sanitari Sant Joan de Déu, Fundació Sant Joan de Déu, Sant Boi de Llobregat, Barcelona 08830, Spain; 10 Universitat de Barcelona, Barcelona 08007, Spain

**Keywords:** clinical mental health research, Delphi survey, priorities, Horizon 2020

## Abstract

Within the ROAMER project, which aims to provide a Roadmap for Mental Health Research in Europe, a two-stage Delphi survey among 86 European experts was conducted in order to identify research priorities in clinical mental health research. Expert consensus existed with regard to the importance of three challenges in the field of clinical mental health research: (1) the development of new, safe and effective interventions for mental disorders; (2) understanding the mechanisms of disease in order to be able to develop such new interventions; and (3) defining outcomes (an improved set of outcomes, including alternative outcomes) to use for clinical mental health research evaluation. Proposed actions involved increasing the utilization of tailored approaches (personalized medicine), developing blended eHealth/mHealth decision aids/guidance tools that help the clinician to choose between various treatment modalities, developing specific treatments in order to better target comorbidity and (further) development of biological, psychological and psychopharmacological interventions. The experts indicated that addressing these priorities will result in increased efficacy and impact across Europe; with a high probability of success, given that Europe has important strengths, such as skilled academics and a long research history. Finally, the experts stressed the importance of creating funding and coordinated networking as essential action needed in order to target the variety of challenges in clinical mental health research.

## 1. Introduction

Mental disorders are among the leading causes of premature death and disability, creating significant social and economic burden in Europe [1,2,3,4,5,6,7,8]. In the last decade, the prevention of mental disorders and the promotion of mental wellbeing have become important areas of focus in the European Union (EU). One of the initiatives of the European Commission, the ROAMER project (Roadmap for Mental Health Research and Wellbeing in Europe), is aimed at gaining more insight into the gaps and needs in the field of mental health and wellbeing. ROAMER covers six major domains—infrastructure and capacity building, biomedicine, psychological research and treatments, social and economic issues, public health and wellbeing—and has the goal of developing a comprehensive and integrated mental health research roadmap orientated to translational research, based on the consensus of all groups of stakeholders and aligned with the policies of the EU’s Horizon 2020 program [9]. However, after the start of the ROAMER project, ROAMER members identified the specific need to establish a cross-sectional task force for gaining more knowledge on the gaps and challenges regarding clinical mental health research, similarly to what had been done for the other domains of mental health research [10,11,12,13,14,15]. The “Clinical Research (CR) Taskforce” was established with the aim of understanding the state of the art in clinical mental health research. One of the initiatives of the CR taskforce was to assess the opinions of experts with regard to gaps and challenges in this field by means of a two-stage Delphi survey. The first step in this Delphi survey was to gather information with open-ended questions in order to study the range of expert opinions [16]. This approach showed that experts give priority to tackling the following challenges: (1) to design and conduct new intervention studies; (2) to understand the diagnostic and therapeutic implications of the mechanisms of disease; and (3) to conduct more research in the field of somatic-psychiatric comorbidity. The second stage of the survey was built on the results of this first stage and aimed to assess the level of consensus with regard to each of the gaps and challenges identified in the first stage. In accordance with the procedures of the NIH Grand Challenges in Global Mental Health initiative [17], in-depth information of the top challenges was also assessed with regard to efficacy, impact and feasibility and to what degree Europe is considered to possess the infrastructure to meet these challenges when compared with other regions of the world. The present paper aims to report the results from this second stage of the Delphi survey and to provide insights into the core consensus-based priorities, the proposed actions and the efficacy, impact, feasibility and unique European qualifications with regard to the identified priorities.

## 2. Methods

### 2.1. The Delphi Approach

The Delphi approach is a technique for collecting data from experts on a subject within their field of expertise [18]. In general, a Delphi survey begins with an open-ended questionnaire investigating experts’ opinions on a certain topic. The structured second stage questionnaire then builds on the results from the first stage [19,20] and assesses the level of consensus regarding the factors identified in the first stage. The Delphi approach has proven its value in providing insight into health-related topics [20,21,22,23].

### 2.2. Second Stage Participants and Procedure

Data for this study were obtained from experts in the field of clinical mental health research who participated in a web-based two-stage Delphi survey aimed at identifying the top priorities in the field of clinical mental health research. The first stage of the survey took place in April, 2013, and aimed at setting priorities for mental health research: the procedure and results of this stage are described in detail elsewhere [16]. The second stage took place in July–September, 2013, and aimed at assessing the level of consensus among the experts with regard to the challenges derived from the first stage. 

All experts (N* =* 313) who were invited to participate in the first stage were invited for participation in the second stage. The expert selection process is described in detail elsewhere [16] (p. 1058), but will be briefly summarized here. Selection was limited to European experts, based on: (1) a minimum h-index of 10 in the Web of Science, or more than 50 publications in the Web of Knowledge, or one of the Top 100 review authors ranked in the Web of Science (years 2007–2011) in the fields of psychiatry, psychology and psychopharmacology; (2) involvement in clinical research and/or European respondents to the Grand Challenges in Global Mental Health survey [17]; (3) European principal researchers conducting clinical research in Europe (selected by two researchers in the field from the databases for clinical research, such as the trial register and the Web of Knowledge); (4) European experts consulted by ROAMER for other surveys who published on clinical research topics in the Web of Knowledge in the last 5 years (CMFC); (5) nominations by the ROAMER Clinical Research Task Force members based on their networks (using the same criteria); (6) nominations by members of ROAMER countries for which recruitment based on the previous criteria resulted in insufficient representation; and (7) ROAMER work package leaders involved in clinical research.

Experts received an email with an individualized survey link to read the informed consent statement and to participate. The link was unique for each participant and could not be forwarded to anyone else. Non-respondents were sent reminder emails (seven reminders were sent in total). This resulted in 86 experts who participated in the second stage of the Delphi survey. WEBROPOL 2.0 [24], an online software program for gathering and analyzing data, was used for this survey. 

### 2.3. Second Stage Questionnaire 

The second stage questionnaire consisted of the following five parts: 

#### Part I: Demographic Variables

The first part of the questionnaire consisted of questions on demographic (*i.e.*, age, gender and country) and professional (*i.e.*, primary affiliation, professional degree, fields of research interests and disorder(s) of expertise) information, consistent with the first stage of the Delphi survey and other ROAMER survey initiatives. 

#### Part II and III: Challenges Built on Pre-Defined Gaps and Open-Ended Questions from the First Stage

The second and third parts of the questionnaire included statements on the importance of the challenges for clinical mental health research (see Table 1). The statements were rated by experts on a 7-point (1, totally disagree; 7, totally agree) Likert scale [25], with a neutral midpoint and the extra possibility to answer “I don’t know”. 

**Table 1:** Level of consensus reached by the experts (N* =* 86) on the statements in Part II and Part III of the questionnaire. IQD, interquartile deviation.

Statements	N	Answered “I Don’t Know” N	Median	IQD
**PART II**				
**Challenge 1:** To develop new, safe and effective treatment interventions (pharmacological, brain related, psychotherapeutic, systemic, psychosocial, eHealth/mHealth approaches and virtual reality/gamification … or a combination of these) is a challenge in the field of clinical research on mental health	86	0	7	1
**PART II**				
**An action to target Challenge 1 is:** To increase research on new intervention approaches in order to gain more insight into their working mechanisms and to successfully develop effective new interventions. This research can/should specifically focus on: - exploring strategies in order to foster adherence to treatments/interventions - exploring mediation factors - exploring the role of these new treatments as an add-on intervention - conducting research in order to identify the best diagnostic measures for complexity (of the interventions) and treatment outcomes - assessing differential treatment effects (are specific approaches more effective for specific subgroups?) - conducting research on eHealth/mHealth approaches and assessing the level of human contact that is needed to motivate individuals towards sustained use of eHealth/mHealth based treatments - conducting research that incorporates patient perspectives in treatment and better trial designs	86	2	6	1
**Challenge 2:** To understand the mechanisms of diseases is a challenge in the field of clinical research on mental health	86	0	7	1
**An action to target Challenge 2 is:** To conduct research in order to reach a clinically relevant understanding of different mechanisms (e.g., psychological mechanisms, biological mechanisms, brain mechanisms, molecular mechanisms and environmental interactions) that may underlie diseases	86	0	7	2
**An action to target Challenge 2 is:** To conduct longitudinal clinical cohort studies with nested randomized controlled trials	86	0	6	2
**Challenge 3:** To evaluate treatment effects is a challenge in the field of clinical research on mental health	86	0	6	2
**An action to target Challenge 3 is:** To conduct research on different approaches to evaluate treatment effects, specifically more research is needed on: - standardization of psycho-therapeutic treatment studies - equivalence trials - side-effects of treatments - alternative and/or non-randomized designs - improved reliability and validity of outcome measures	86	0	6	2
**An action to target Challenge 3 is:** To increase the involvement of health-care staff, among others in order to stop non-effective treatments	86	0	6	2
**Challenge 4:** To perform proof of concept clinical trials for innovative treatments is a challenge in the field of clinical research on mental health	86	4	6	2
**An action to target Challenge 4 is:** To identify or develop standard definitions and guidelines to increase the understanding of the term ‘proof of concept’	86	3	5	1
**Challenge 5:** To gain insight into the role of comorbidity between mental disorders and somatic conditions for diagnosis, treatment decisions, and treatment and patient-related outcomes is a challenge in the field of clinical research on mental health	86	0	6	2
**An action to target Challenge 5 is:** To develop research in order to better understand mechanisms of comorbidity and how to investigate and treat comorbidity (including diagnostic strategies)	86	0	6	2
**An action to target Challenge 5 is:** To increase research on intervention studies that target comorbidity	86	0	6	2
**Challenge 6:** To improve diagnostic strategies and the stratification of diseases is a challenge in the field of clinical research on mental health	86	0	6	2
**An action to target Challenge 6 is:** To define and validate stages for different diseases	86	0	6	3
**An action to target Challenge 6 is:** To develop and validate new diagnostic approaches and to foster standardization of diagnostic tools	86	0	6	2
**Challenge 7:** To improve interventions in terms of return/to/work, presenteeism-absenteeism is a challenge in the field of clinical research on mental health	86	2	6	2
**An action to target Challenge 7 is:** To target work disability and return-to-work as the main outcomes	86	2	5.5	3
**An action to target Challenge 7 is:** To identify or develop standard measures for return-to-work and related outcomes	86	2	6	3
**Challenge 8:** To determine the cost/effectiveness of interventions to increase rates of return to work, presenteeism, decreased rates of absenteeism is a challenge in the field of clinical research on mental health	86	3	6	2
**An action to target Challenge 8 is:** To foster the standard inclusion of cost-effectiveness assessments in intervention studies	86	2	5	1
**Challenge 9:** To overcome methodological gaps regarding the inclusion of patient preferences by study designs is a challenge in the field of clinical research on mental health	86	3	5	2
**An action to target Challenge 9 is:** To develop or identify standard measures of preferences (thus: to reach more consistency in the measures of preference used)	86	6	5	2
**An action to target Challenge 9 is:** To develop designs for preference studies	86	6	5	2
**Challenge 10:** To overcome methodological gaps regarding psychotherapeutic interventions studies (a specific gap is for instance that placebo studies are missing) is a challenge in the field of clinical research on mental health	86	1	6	2
**An action to target Challenge 10 is:** To explore what is the most likely accepted placebo in psychotherapeutic studies	86	2	5.5	2.75
**An action to target Challenge 10 is:** To increase research on the process and outcomes of different psychotherapies, especially by conducting more RCT’s in this field	86	2	6	2.75
**Challenge 11:** To overcome methodological gaps regarding psychopharmacological intervention studies (one of the specific gaps is that most pharmacological studies are funded by the pharmaceutical industry and that the pharmaceutical industry is withdrawing from psycho-pharmacological research altogether: leaving a gap in research) is a challenge in the field of clinical research on mental health	86	0	6	2
**An action to target Challenge 11 is:** To work more closely with industry in a precompetitive environment to ensure high quality trial design, on an independent basis	86	3	6	2
**A general action for targeting all gaps:** Increasing funding/financial investment in order to conduct more research is a general action that is relevant for most challenges mentioned	86	3	7	1
**A general action for targeting all gaps:** To establish (European) research networks to coordinate and facilitate clinical research is a general action that is needed in order to reach most of the challenges mentioned	86	0	6.5	1.25
**PART III**				
**Challenge 12:** To conduct research on prevention approaches is a challenge in the field of clinical research on mental health	86	0	6	2
**Challenge 13:** To gain more insight into the best outcomes to use (including the use of alternative outcomes) is a challenge in the field of clinical research on mental health	86	1	6	1
**Challenge 14:** To understand intervention processes and mechanisms is a challenge in the field of clinical research on mental health	86	0	6	2
**Challenge 15:** To conduct research on how to overcome stigma and social exclusion is a challenge in the field of clinical research on mental health	86	0	6	2.25
**Challenge 16:** To foster the delivery of and access to mental healthcare is a challenge in the field of clinical research on mental health	86	1	6	2
**Challenge 17:** The translation, integration and dissemination of research findings and treatments is a challenge in the field of clinical research on mental health	86	0	6	2
**Challenge 18:** Conducting research is a challenge in the field of clinical research on mental health	86	2	6	2
**Challenge 19:** Facilitating research is a challenge in the field of clinical research on mental health	86	3	6	2

The eleven statements of the second part of the questionnaire were derived from the results of the closed-ended questions about the pre-defined gaps/challenges presented in the first stage and were supplemented with twenty statements on potential actions/advances needed to meet these challenges. 

The eight statements included in the third part of the questionnaire were derived from qualitative analyses of free-text responses in the first stage and were supplemented with a list of 10–15 actions/advances needed per challenge (as identified in the first stage). The experts were asked to select the three most important actions/advances needed per challenge (in Part III).

#### Part IV: Selection of Top 2 Priorities

The fourth part included one question asking the experts to select the two most important challenges from the total of 19 challenges presented in Part II and Part III. 

#### Part V: Efficacy, Impact, Feasibility and European Strength

The fifth part asked the experts to elaborate on the top 2 challenges of their choice in terms of the following four parameters:

1. Efficacy,* i.e.*, the likelihood that addressing the challenge(s) would result in an effective intervention to diminish the appearance of a disease or its consequences or solve a concrete problem.
2. Impact on Europe,* i.e.*, the probability that targeting the challenge(s) would result in an impact for Europeans and/or for society (e.g., a decrease in disease burden, improvement of wellbeing, economic benefits,* etc.*).
3. Feasibility in Europe,* i.e.*, can the challenge(s) and actions be addressed in Europe?
4. European research strength,* i.e.*, relative competitiveness of Europe to other regions to meet the challenge and to achieve the advances.

### 2.4. Second Stage Analyses

Descriptive analyses were conducted to describe sample characteristics. Median scores were calculated per statement in order to characterize the answer category above and below which 50% of the answers fell. Interquartile deviations (IQDs), which form the distance between the 25th and the 75th percentiles, were used to represent the spread of the data and to assess the level of expert consensus per statement. A smaller IQD value indicates a small data spread: an IQD ≤1 is considered to be good consensus on a 7-point scale, meaning that 50% of all cases fall within 1 answer category from each other [26]. 

The median scores also indicated whether the experts disagreed (median score ≤ 3) or agreed (median score ≥ 5) with the consensus statement, with a score of 4 indicating neutrality. The answer category ‘I don’t know’ was not included in the consensus calculation, but was analyzed separately and presented in Table 1. Table 1 also includes for each statement: (1) the number of experts who rated the statement; (2) the median score; and (3) the IQD score. 

Moreover, only when consensus was reached on a challenge in this second stage of the survey, the first stage answers were consulted to describe the sub-challenges underlying the main challenge. It should be noted that there were no consensus assessments for the sub-challenges, as this would have made the questionnaire too extensive and would have formed a time burden for the experts.

Finally, the responses to the open-ended questions on efficacy, impact, feasibility and European strength (requested only for the top 2 challenges selected) were categorized by one of our researchers (items were grouped into answer categories) and checked by a second researcher. 

## 3. Results 

### 3.1. Sample Characteristics

Of the invited experts, 86 (78% males) participated in the second stage of the Delphi survey. The respondents represented different countries in Europe, with overrepresentation of the United Kingdom (25%), Netherlands (22%) and Italy (11%). The experts represented various clinical research areas (*i.e.*, clinical trials, epidemiology/public health research, basic research, psychological sciences and patient care), had different professional degrees (78% professors; 21% MD/PhD; 1% MSc) and worked at different types of institutions (47% at a university or a teaching hospital and 42% at a non-profit university). Moreover, the experts collectively represented expertise on various disorders (*i.e.*, depression/bipolar disorders (60%), psychotic disorders (43%), anxiety and PTSD (30%), comorbidity between mental and somatic disorders (20%), substance use and abuse (16%), child and adolescent psychiatric disorders (14%), personality disorders (7%), somatoform disorders (5%) and eating disorders (3%)). 

### 3.2. Top Priorities

Table 1 shows that consensus (IQD ≤ 1) and agreement (answer ≥ 5) were reached on the importance of three challenges:

1. The development of new, safe and effective treatment interventions;
2. Understanding the mechanisms of diseases; and
3. Gaining more insight into the best outcomes to use for clinical mental health research.

#### Priority 1: The Development of New, Safe and Effective Treatment Interventions 

The statement with regard to this challenge had a median score of seven (full agreement) and an IQD of one, indicating expert consensus. The specific sub-challenges underlying this main challenge were derived from the first stage results, namely:

- increasing the utilization of tailored approaches (personalized medicine);
- developing (eHealth/mHealth) decision aids/guidance tools that help the clinician to choose between various treatment modalities based on the type of mental disorder, stage/progression, previous outcomes, comorbidity and other factors;
- the development, assessment and use of eHealth/mHealth tools for the patient;
- developing specific treatments in order to better target comorbidity;
- developing more specific pharmacological, psychological and somatic treatments;
- increased attention for combined psychological and psychopharmacological approaches;
- developing deep brain stimulation, transcranial magnetic stimulation and other neuro-stimulation approaches for (treatment refractory) mental disorders.

Consensus was also reached on the actions needed to address this challenge (median score of six and IQD of one), namely:

□ To stimulate research on new intervention approaches yielding insight into the working mechanisms and to successfully develop effective new interventions. This research can/should specifically focus on:
  ▪ exploring strategies in order to foster adherence to treatments/interventions;
  ▪ exploring mediating factors;
  ▪ exploring the role of these new treatments as an add-on intervention to existing interventions;
  ▪ conducting research in order to identify the best diagnostic measures for complexity of interventions and treatment outcomes;
  ▪ assessing differential treatment effects: are specific approaches more effective for specific subgroups?
  ▪ conducting research on eHealth/mHealth approaches and assessing the level of human contact that is needed to motivate individuals towards sustained use of eHealth-/mHealth-based treatments in blended eHealth models;
  ▪ conducting research that incorporates patient perspectives in treatment and in trial designs.

#### Priority 2: The Challenge to Understand the Mechanisms of Diseases

The statement on this challenge showed a median score of seven and an IQD of one, indicating expert consensus on this challenge in the field of clinical mental health research. Again in-depth analyses of the first stage answers were used to gain more insight into the specific sub-challenges, which were:

- gaining more insight into the neurobiological base of brain disorders;
- understanding staging and sub-typing of clinical trajectories based on underlying mechanisms of disease;
- exploring underlying mechanism in co-morbidity in severe mental disorders;
- taking into account protective factors for the development or course of disease;
- acknowledging the developmental perspective, in order to be able to intervene early;
- identifying environmental risk factors and their role in the mechanism of disease;
- integrating biological research with epidemiological, psychological and genetic research; and
- studying specific populations, such as children, adolescents and older people.

Although the experts did agree on the importance of the challenge to understand the mechanisms of disease, there was no expert consensus for the two actions suggested for meeting this challenge (as indicated by an IQD score of two): ‘to conduct research in order to reach a clinically relevant understanding of different mechanisms’ and ‘to conduct longitudinal clinical cohort studies with nested randomized controlled trials’. 

#### Priority 3: The Challenge to Gain More Insight into the Best Outcomes to Use for Clinical Mental Health Research

The statement on this challenge resulted in a median score of six and an IQD of one, indicating agreement and expert consensus. Several specific sub-challenges with regard to this main challenge were collected from the results of the first stage:

- standardizing research outcomes of psychological interventions;
- identifying outcome related biomarkers;
- increasing the focus on reducing long-term chronicity; and
- personalized prescribing of antipsychotic medication, discriminating between patients who do and patients who do not require sustained use of medication.

The experts were asked to select three actions needed to address this challenge. The following actions were most often selected:

□ Building a system of routine outcome measures (ROM) in clinical practice;
□ Conducting effectiveness/efficacy/cost-efficacy studies;
□ Including non-medical measures that are relevant for patients as outcomes of intervention research, reflecting personal and social recovery (e.g., empowerment, income, housing, work life status, sense of meaningful life);
□ Conducting (alternatives for) randomized controlled trials (RCTs).

**Figure 1:**
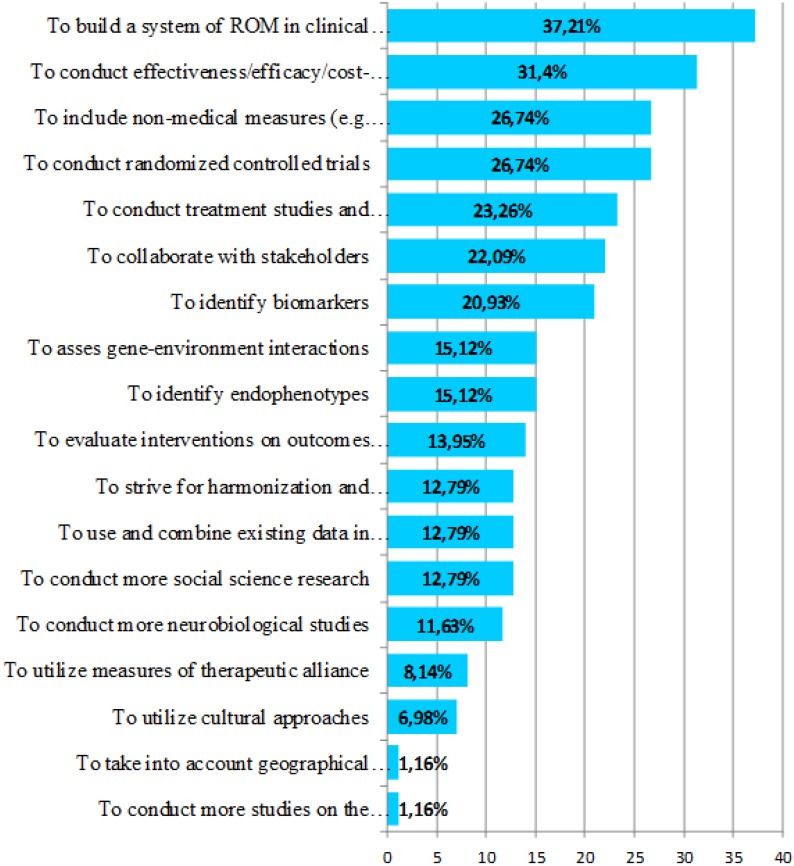
Experts’ selection of actions to meet the challenge “to gain insight into the best outcomes to use”. The percentage indicates the proportion of expert respondents that selected the action.

Figure 1 shows all of the actions presented to the experts, as well as the percentage of experts selecting an action.

### 3.3. Efficacy, Impact, Feasibility and European Strength Concerning the Top Two Priorities 

**Figure 2:**
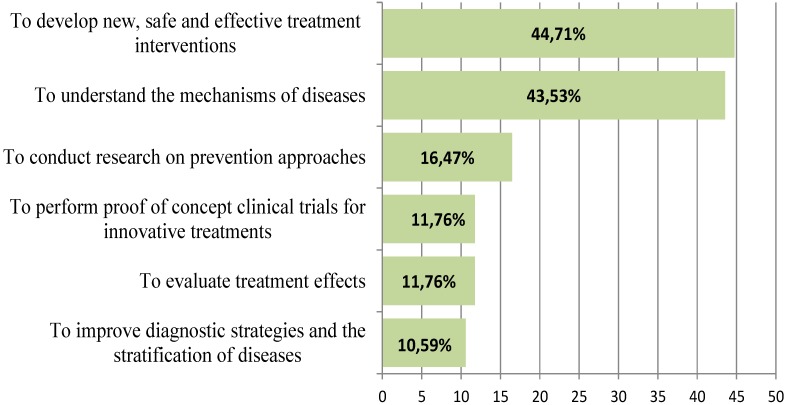
Percentage of experts that selected a challenge as the main priority in the field of clinical mental health research. Note: this figure only presents the challenges chosen by >10% of the experts.

The open-ended question asking the experts to select their top two priorities in the field of clinical mental health research showed that “to develop new, safe and effective treatment interventions in the field of clinical research on mental health (Priority 1)” and “to understand the mechanisms of diseases (Priority 2)” were most often chosen as priorities. Figure 2 presents the challenges that were chosen by more than 10% of the experts. 

#### 3.3.1. Importance of the Top Two Priorities in Terms of Efficacy

In terms of efficacy, the experts indicated that meeting the challenge of developing new, safe and effective interventions will: (1) result in fewer symptomatic people; (2) help to find effective treatments; (3) result in a higher likelihood of response; (4) optimize mental health by discontinuation of approaches that have no clinical benefit; (5) reduce the burden of mental disorder; (6) result in greater therapeutic effectiveness; (7) result in less side effects; and (8) result in affordable mental healthcare.

The challenge of understanding the mechanisms of diseases is thought to: (1) result in the development of new effective treatments that will result in the reduction of disease prevalence and optimizing disease outcome; (2) foster the development of specific diagnostic tools and personalized treatments; (3) improve the classification of diseases; and (4) optimize prevention approaches.

#### 3.3.2. Importance of the Top Two Priorities in Terms of Impact

In terms of impact, the experts indicated that meeting the challenge of developing new, safe and effective treatments will: (1) decrease disease burden and improve wellbeing, thus providing substantial benefits given the current rate and burden of mental disorders; (2) result in better quality of life for patients and their relatives; and (3) result in more productivity at work (economic benefit). 

Meeting the challenge of understanding the mechanisms of diseases will, in turn, establish the bases to: (1) develop better treatments; (2) improve patient’s quality of life; (3) decrease the incidence of diseases (by defining preventative measures) and mortality rates associated with mental disorders (by preventing the increased risk of premature death either by suicide or by natural causes derived from the comorbid physical conditions associated with a particular mental disorder); and (4) refine current interventions.

#### 3.3.3. Feasibility and European Strength Regarding Targeting the Top Two Challenges

In terms of feasibility, the experts indicated that both challenges are feasible and can easily be achieved in Europe, but that specific funding is necessary. European strengths highlighted by the experts that can be useful to address these two challenges are: (1) a large pool of skilled academics; (2) long research history; (3) well-trained mental health professionals; (4) existing national clinical research networks; (5) European collaborative networks; and (6) expertise and research infrastructure.

## 4. Discussion

The present study aimed to assess the level of expert consensus regarding gaps and challenges in the field of clinical mental health research, in order to identify the themes of the top priorities that need to be addressed in the following 10–15 years. 

The first stage results of the expert survey (discussed in [16]) highlighted the priorities to develop new interventions, to understand the mechanisms of disease and to conduct more research in the field of somatic-psychiatric co-morbidity. The present study further supported the significance of developing new, safe and effective interventions and understanding the mechanisms of disease by revealing a high level of expert-consensus regarding their importance. Moreover, they were also most often chosen as the top two challenges by experts. The present study also provided further insight as to how meeting these challenges may play a role with regard to efficacy (*i.e.*, the likelihood that addressing the challenge(s) would result in an effective intervention to diminish the appearance of a disease or its consequences or solve a concrete problem) and impact (*i.e.*, the probability that addressing the challenge(s) would result in an impact for Europeans and/or for society, e.g., a decrease in disease burden, improvement of wellbeing, economic benefits,* etc.*) in Europe. 

### 4.1. Development of New Interventions 

The experts were of the opinion that the development of new effective interventions will have several results, namely fewer symptomatic people, guidance towards finding more effective treatments, less overtreatment in those who do not benefit, the reduction of medication side-effects, the reduction of the burden of mental disorders and more affordable mental health services. Moreover, the experts pointed out that the development of new effective interventions will have a substantial impact on European society due to the current high prevalence and burden of mental disorders in Europe, as this will lead to a decrease of disease burden (if the treatments can ameliorate the symptoms of or eventually cure the disorders), a better quality of life for patients and their relatives and increased productivity at work (resulting in economic benefits). These expectations indicate that the experts perceive addressing this challenge as an important first step towards solving important themes in the field of mental health, such as the economic and health-related burden caused by mental disorders [1,3,5,6] and the problem of side-effects of treatments, which form an important barrier with regard to treatment adherence and patient satisfaction (for a review, see [27]). 

Reaching the development of new interventions in the field of mental health clinical research can only be achieved by targeting the specific sub-challenges on which this challenge is built. One of the specific sub-challenges underlying the development of new interventions according to the experts is the development, assessment and use of (blended) eHealth/mHealth interventions, which appears to be consistent with the (more detailed) priorities identified in previous research [14,28]. In the last decade, the use of eHealth/mHealth strategies in the field of mental health has become an important topic of interest [10,29,30]. Recent advances in eHealth/mHealth support the potential of Internet-delivered interventions by showing positive treatment effects and lower costs [31,32,33]. Computerized cognitive behavioral therapy and online social forums are examples of such treatments [32,34]. Moreover, eHealth/mHealth strategies have the ability to foster the accessibility of care, interactivity, patient engagement and flexibility in terms of standardization and personalization [35,36]. Yet, there is still much to gain with respect to the adoption and implementation of eHealth/mHealth interventions. Although professionals are interested in utilizing eHealth/mHealth approaches, the implementation process is slow and subject to barriers, such as lack of experience and knowledge [37,38,39,40]. Conducting better evaluation methods, such as RCTs and studies on the predictors of adoption, adherence, implementation and success factors of eHealth/mHealth interventions, are recommended in order to foster the use and implementation of eHealth/mHealth [31]. 

The utilization of personalized medicine approaches, which are built, similarly to the traditional model of mechanistically targeted and individualized psychological interventions, on the idea that a patient’s unique characteristics determine disease susceptibility as well as treatment response [12,41], forms another challenge in the way towards the development of new successful interventions. Personalized medicine has shown successes in the field of general medicine, but is not yet established in the field of mental health [42]. Personalized medicine is, among others, expected to be promising when gaining insight into genetic vulnerability or protective factors. A recent review regarding personalized medicine describes the information on characteristics that can be gathered and used for tailoring interventions in psychiatry [43], such as genetic and epigenetic changes. Though personalized medicine is not yet fully implemented in psychiatry and still is at an information-gathering phase [43], the experts from our survey indicated a need to focus more on this tailored approach in the field of mental health in the upcoming 10–15 years. Behavioral psychotherapies are by their very nature and models ‘personalized’ and can be seen as tailored therapies (see, e.g., [12,13]). Moreover, psychiatry may be in need of interventions based on self-monitoring and self-management (including self-titration of medication), thus moving away from exclusive reliance on guidelines for average patients and average treatment effects. A recent study on this approach in hypertension has shown that it results in better results than traditional guideline-based treatments [44]. Comparable initiatives may be interesting for further testing in psychiatry, and thus, conducting RCTs and studies on such approaches is highly recommended for the future. 

Another topic regarding the development of new interventions that was mentioned by the experts was the development of decision aids/guidance tools for professionals. Unfortunately, no specific information was obtained on the kind of guidance tools that are needed. Guidance tools can serve many purposes, and they can even be combined with the challenge of developing eHealth/mHealth and personalized medicine interventions. For instance, guidance tools can be embedded in an eHealth/mHealth format and/or they can be developed in order to foster the use of personalized medicine approaches by professionals. The effectiveness of such an approach was established in the treatment of major depressive disorder in the primary care setting [45] and the mental health setting [29]. Guidance tools for professionals can also be developed in order to foster patient involvement (a goal that was clearly derived from the first stage results, but on which no clear consensus was reached in the second stage results). As also discussed in the first stage manuscript [16], shared decision making (SDM) is an approach in which patient centeredness is promoted and which may enhance patient adherence to treatment. SDM has received growing attention in the last decade and is perceived as the way to go when more than one option can be chosen [46]. Yet, patients, as well as professionals have difficulties with implementing the SDM approach [47,48]. Developing and imbedding guidance tools can be helpful in fostering SDM, and with that, patient involvement and is, therefore, recommended for the future. A recent pilot of SDM in combination with routing outcome monitoring (ROM) in patients with somatic symptom disorders has already shown the good adherence of patients, as well as professionals and the improvement of symptom scores [49].

A final sub-challenge that could be categorized in the top challenge of developing new interventions was the development of new specific interventions. Topics of interest were the development of deep brain stimulation therapies for the treatment of refractory mental disorders, developing more specific treatments (*i.e.*, pharmacological, psychological and somatic) and treatments for targeting co-morbidity, as well as increasing attention for interactive psycho-pharmacological approaches and increasing the development and evaluation of transcranial magnetic stimulation approaches. Development and study on these approaches is therefore essential for the upcoming years. 

### 4.2. Understanding the Mechanisms of Diseases

The challenge of understanding the mechanisms (e.g. relevant causal factors as well as relevant moderators and mediators) of disease is also expected to have a great influence with regard to efficacy and impact. According to the experts, addressing this challenge is essential to seek clues for the development of new effective treatments that will result in diminishing the disease and its consequences. Furthermore, increasing knowledge on the mechanisms underlying mental disorders will foster the development of specific diagnostic tools and personalized treatments, improve the classification of diseases and optimize prevention approaches. Improving our understanding of the mechanisms of disease is thus related to the previous challenge of developing new and effective interventions and is, therefore, expected to have a similar impact in terms of leading to better refined treatments, better quality of life for patients and their relatives, a decrease of the incidence of diseases (by defining and conducting preventative measures) and a decrease in mortality rates associated with mental disorders. The latter expectation is very important and can be supported as follows. It is known that people suffering from mental disorders are at-risk of premature death due to non-natural causes, such as suicide and accidents, as well as natural causes, such as cardiovascular and respiratory diseases [50,51,52,53,54]. Achieving deeper knowledge of the mechanisms of mental disorders and of the co-morbidity between mental and physical conditions can result in the ability to tackle those mechanisms through biological or psychological interventions and, consequently, prevent early mortality due to natural causes. Furthermore, understanding at which stage in the development of a particular mental disorder a patient is more likely to attempt suicide will foster the ability to prevent it. For instance, a better understanding of depression might help not only to treat depression, but also to reduce the time of exposure to suicide risk. It is, therefore, essential to address this challenge of understanding the mechanisms of disease. An elaborate discussion of the associated sub-challenges that need to be tackled can be found elsewhere (see [16]). Promoting that discussion, we want to add that an integrated approach encompassing the knowledge of the mechanisms of disease may give new insights into staging, diagnosis and treatment interventions of mental disorders, enhance early detection and early intervention and provide us with possibilities to improve resilience. This experts’ advice is consistent with the Research Domain Criteria (RDoC) initiative of the National Institute of Mental Health and the European traditional emphasis on multilevel and multimodal assessments, which aims to prioritize mental health research based on constructs derived from an identified underlying neurobiology, rather than consensus-based diagnostic entities [55]. The RDoC initiative may be valuable to foster basic research, but has been criticized for lacking clinical transference [56]. 

### 4.3. Insight into the Best Outcomes to Use in Clinical Mental Health Research

The third priority on which expert consensus was reached is to gain insight into an improved set of broader outcome measures to evaluate the effect of clinical interventions in several domains, including alternative outcomes. Traditional outcome measurement in the field of mental health has mainly focused on the assessment of psychopathological outcomes, while more recent initiatives also include a broader range of outcomes, such as patient motivation, work-related outcomes and quality of life [57,58,59,60,61,62]. Broad outcome measurement and monitoring are important, because these can provide detailed information on the functioning of health services and on how to better implement and provide interventions and services in the health care system. Further, both professionals and patients may benefit from proper outcome measurement,* i.e.*, with regard to treatment planning and treatment monitoring. According to our experts, building a system of routine outcome measures in clinical practice is perceived as the most important action needed to meet this challenge. Recently, routine outcome monitoring has become important in the field of mental health [63,64,65]. Yet, professionals face difficulties with its implementation [66], for instance due to the burden of completing multiple questionnaires. A recent study [67] explored professionals’ experiences with an online routine outcome monitoring tool that included online questionnaires and feedback on the scores. Moreover, the paper-based questionnaires could be entered into the online tool by a research assistant. Results showed that the online approach alone was not sufficient for fostering structural monitoring, but that the availability of a helping research assistant seemed essential for the feasibility of the online tool. Furthermore, the professionals indicated the need for being trained on interpreting the feedback from the scores provided by the tool. Further insights into how to foster routine outcome monitoring is therefore recommended. Besides the importance of routine outcome monitoring, the results of our expert survey indicated that standardization, which is beneficial when professionals and patients, for instance, use different services, is one of the sub-challenges underlying the main challenge on defining outcomes in clinical mental health research. Other specific challenges were to identify outcome-related biomarkers and to increase the focus on reducing long-term chronicity. In line with the experts’ opinions, we recommend the utilization of effectiveness/efficacy/cost-efficacy studies and RCTs to gain more insight into the best outcomes to use. 

### 4.4. Strengths and Limitations of This Study

The strengths of the present study include that the sample of experts represented nineteen European countries, various clinical research areas, different types of institutions and expertise on several disorders. This underlines the high generalizability of the study findings. Further, open-ended questions were used to collect in-depth information on the two challenges forming the top priorities in terms of efficacy, impact, feasibility and European strength. Nonetheless, there may be some limitations as well. It should be noted that only 28% of the experts responded to our invitation: this low response rate may bias the result. However, when considering only the experts that were involved in the first stage of the consultation (N *=* 89), the response rate of the second wave is about 96%. This suggests that only a few experts were lost to follow-up, which is, in general, seen as a strength in Delphi surveys [68]. Next, the second stage questionnaire did not include consensus assessments with regard to the sub-challenges, but for the main challenges only. Assessing the consensus for all sub-challenges would have specified which sub-challenges are perceived as a priority. However, utilizing this approach would have resulted in a quite extensive questionnaire, forming a significant time burden for the experts. Another potential limitation is that the opinions of highly experienced experts may have been underestimated compared to less experienced ones, as the opinions of all experts were equally weighted in the analyses. However, our strict criteria for the selection of participants and our final sample characteristics (indicating that most of the experts were professors) ensure that the experts are indeed sufficiently experienced.

## 5. Conclusions 

The present study identified core priorities for the upcoming 10–15 years in the field of clinical mental health research based on European experts’ consensus ratings. Moreover, the study pointed to specific sub-challenges that need to be addressed on the way towards meeting the core priorities. It also adds synergistically to more in-depth explorations provided by ROAMER [12,14,15]. The experts indicated that the top priorities are feasible in Europe, and they underlined that Europe has the strength to face these challenges. Specifically, the experts mentioned the highly skilled academics, long research history, well-trained mental health professionals, existing national clinical research networks, European collaborative networks and expertise and research infrastructure. However, it should be noted that, although the experts consider Europe to have important strengths to meet these challenges, they stress the importance of funding possibilities for clinical mental health research, for example in the EU Horizon 2020 program, and in national funding agencies. 
